# Initial Clinical Experience with the Biodegradable Absnow^TM^ Device for Percutaneous Closure of Atrial Septal Defect: A 3-Year Follow-Up

**DOI:** 10.1155/2021/6369493

**Published:** 2021-07-30

**Authors:** Yifan Li, Yumei Xie, Boning Li, Zhaofeng Xie, Junjun Shen, Shushui Wang, Zhiwei Zhang

**Affiliations:** ^1^Department of Pediatric Cardiology, Guangdong Cardiovascular Institute, Guangdong Provincial People's Hospital, Guangdong Academy of Medical Sciences, Guangdong Provincial Key Laboratory of South China Structural Heart Disease, Guangzhou 510100, China; ^2^Department of Pediatric Cardiology, Shenzhen Children's Hospital, Shenzhen 518038, China

## Abstract

**Objective:**

We reported the 3-year follow-up results of initial clinical experience with the Absnow^TM^ device, a novel biodegradable occluder for percutaneous closure of atrial septal defect (ASD).

**Background:**

The Absnow^TM^ device is a total biodegradable septal occluder with double-disc poly-L-lactic acid (PLLA) framework and PLLA membranes intergraded into the device to ensure its biodegradability, clinical safety, and efficacy.

**Methods:**

Five pediatric patients were enrolled from May to June 2018 in our institution and were followed up for 3 years. A clinical evaluation and transthoracic echocardiography were performed at 24 hr, 1 month, 3 months, 6 months, 12 months, and yearly after implantation. Primary endpoints were a composite clinical success, comprising of clinical closure success and safety at the 36-month follow-up evaluation. Secondary endpoints included technical success, procedure success, closure success, and safety at each of the follow-up visits.

**Results:**

The median subject age was 3.6 years (range 3.1–6.5 years). The mean ASD diameter was (13.7 ± 2.9) mm. The median device size was 20 mm (range 14 to 24 mm). Technical and procedure success was achieved in 100% (5/5) of the patients. At 2-year follow-up, 3 of the 5 patients developed new-onset residual shunts and 2 of them reached a moderate degree. At 3-year follow-up, the residual shunt size increased over time in all the 3 patients, and 1 of them had right ventricular enlargement. All of the 5 patients were free from serious adverse events during the 3-year follow-up, with no device embolization, thromboembolization, or reintervention to the target defect.

**Conclusion:**

This 3-year follow-up result of initial experience with the biodegradable Absnow^TM^ device has demonstrated acceptable safety with no procedural complications. Notably, the high rate of residual shunt significantly affected its efficacy. The long-term safety and efficacy of the device should be further evaluated in a large cohort of patients in future studies.

## 1. Introduction

Secundum atrial septal defect (ASD) is a common cardiac congenital anomaly accounting for about 10% of cases of congenital heart disease. Nowadays, percutaneous device closure has become the preferred method over the surgical repair for secundum ASD with the improvement of design concepts and materials discoveries over time.

Biodegradable devices have emerged as an alternative to a permanent metal prosthesis to prevent potential late complications such as erosion or perforation of the device, thrombus formation, frame fractures, and nickel allergy [1–4]. They provide a temporary occlusion function that utilizes endogenous healing to achieve a complete sealing of the defect, before being absorbed and replaced by normal tissue. The BioStar device (NMT Medical, Boston, MA) was the first partially biodegradable occluder approved for ASD and patent foramen ovale closure in humans [5–7]. Although it was withdrawn from the market mainly due to its safety issues attributable to the biological material and immunological properties of the device, it represented a huge step toward the clinical application of biodegradable occlusion devices.

The use of completely biodegradable septal defect occlusion devices in an experimental setting has been reported. The representative materials used in biodegradable devices are PLLA, poly(D, L-lactic acid) (PDLLA), poly-D-L-lactide-glycolide, polydioxanone, and poly(*ε*-caprolactone) (PCL) [8–11]. PLLA is reported to have excellent biocompatibility and bioresorbability in the human body and has been used in a number of biomedical applications including implants, drug delivery, and tissue engineering [12–14]. Our previous preclinical study demonstrated that in a swine ASD model implanted with a PLLA septal occluder, endothelialization was mostly completed at 3 months [15], and almost complete degradation of the device was shown at 36 months [16].

The Absnow^TM^ ASD closure system (Lifetech Scientific, Shenzhen, China) is a novel, biodegradable septal occluder designed for the percutaneous closure of ASD. The framework and membranes of the device are made of PLLA, which makes it totally biodegradable. Our previous report has shown the preliminary efficacy and safety of this device in initial clinical evaluations with high levels of short-term clinical success [16]. In the present study, we report for the first time the long-term efficacy and safety of the initial experience with the Absnow^TM^ device in 5 pediatric patients.

## 2. Materials and Methods

### 2.1. Study Design

A prospective, nonrandomized, 5 patients pilot study was started between May and June 2018. Patients (age ≥3 years old, weight ≥10 kg) who were scheduled to undergo percutaneous ASD closure at our institution were screened for inclusion in the study. The study protocol was approved by our institutional review board and was undertaken in accordance with the Declaration of Helsinki. Written informed consent was obtained from the patient's legal representative prior to subject enrollment. Clinical evaluations including physical examination, electrocardiography (ECG), and transthoracic echocardiography (TTE) were performed before the procedure and were repeated 24 hr after the procedure and at 1, 3, 6, 12, 24, and 36 months.

### 2.2. Patient Selection

Inclusion criteria were a secundum ASD of ≥5 mm and ≤30 mm by TTE measurement, adequate septal rims for device stability (superior to the mitral valve by 7 mm, superior to the coronary sinus, superior/inferior vena cava, and pulmonary vein by 5 mm), and signs of right ventricular volume overload and/or evidence of significant left-to-right shunting (Qp : Qs ≥ 1.5 : 1) [16]. Exclusion criteria included concomitant congenital heart defect or comorbidity which could contribute to additional intervention during the study period. Patients with other comorbidities, including a history of pulmonary hypertension, infective endocarditis, intracardiac thrombi, uncontrolled arrhythmia, pregnancy, contraindication to antiplatelet therapy, allergy to PLLA materials, and history of ASD repair or left atrial appendage occlusion, were also excluded from the study.

### 2.3. Study Device

**Figure 1:**
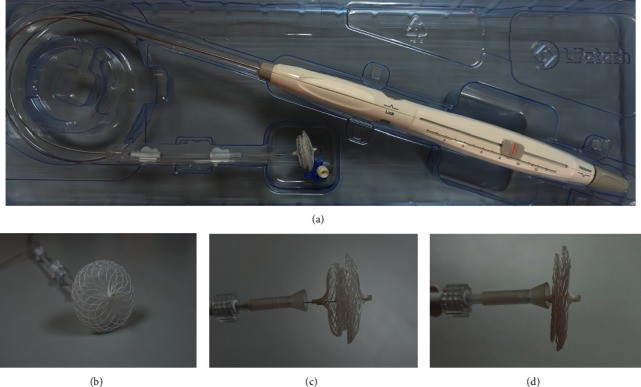
The Absnow™ ASD closure system and the structure of the PLLA device. (a) The integral structure of the Absnow™ ASD closure system. (b) Frontal view of the PLLA device. (c) The unlocked state of the PLLA device. (d) The locked state of the PLLA device.

**Figure 2:**
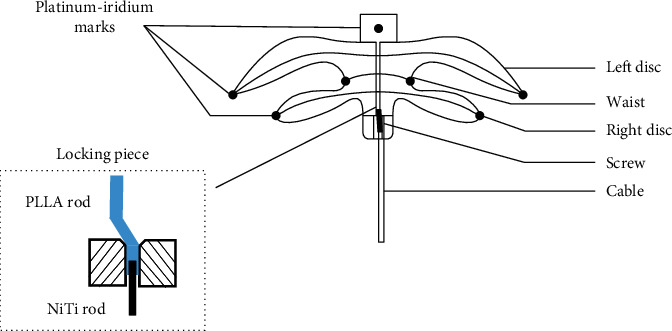
Schematic representation of PLLA device design. The seven platinum-iridium marks are shown on the device. A PLLA rod is connected to a NiTi rod to form the main structure of the locking piece.

The Absnow^TM^ ASD closure system consists of the PLLA device and the delivery system (Figure 1(a)). The device is composed of a double-disc PLLA framework bonded with three pieces of PLLA membrane sewn into two discs and the waist and can be in the “unlocked” or “locked” state by control of the locking system (Figures 1(b)–1(d)). The device is available in waist sizes from 6 to 32 mm at 2 mm increment deliverable through 8–14 Fr sheaths. For devices between 6 and 10 mm, the left atrial disc is 12 mm larger than the waist and the right atrial disc is 8 mm larger. For devices between 12 and 32 mm, the left atrial disc is 14 mm and the right atrial disc is 10 mm larger than the waist, respectively. Seven platinum-iridium marks are fixed in the membrane and the tip of the device to render its visibility as the PLLA framework and membrane are invisible on fluoroscopy (Figure 2). The device is attached by a locking piece (Figure 2) onto its delivery system consisting of a delivery cable and a controlling handle. The delivery system allows for device locating, repositioning, or retrieval in an “unlocked” state and final device release in a “locked” state.

### 2.4. Study Procedure

The closure procedure was performed with fluoroscopic and TTE guidance under general anesthesia. All patients received intravenous heparin (100 IU/kg) and prophylactic antibiotic (cefazolin 50 mg/kg) prior to device implantation.

**Figure 3:**
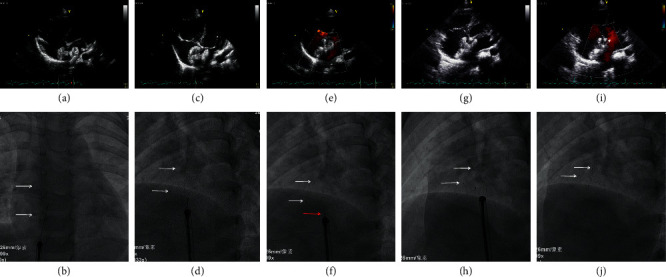
The deployment process of the PLLA device under TTE and fluoroscopic guidance. (a-b) The left disc was unfolded under TTE, and the seven marks were shown on fluoroscopy (white arrows). (c-d) The right disc was fully opened under TTE. White arrows indicated the position of the seven marks. (e-f) The right disc was pushed towards the atrial septum under TTE. Notice the position of the seven marks (white arrows) and the NiTi rod of the locking piece (red arrow). (g-h) The position and shape of the device were evaluated by TTE, and the NiTi rod was detached from the device on fluoroscopy. White arrows indicated the seven marks. (i-j) After the device was released, the final position and shape of the device were evaluated by TTE and fluoroscopy. White arrows indicated the seven marks.

Vascular access was obtained from the right femoral vein and a complete hemodynamic evaluation was performed. The size and anatomy of the defect were determined by 2-dimensional (2D) and color Doppler TTE in multiple planes during the procedure. Transesophageal echocardiography (TEE) was not used in our study since the defect size could be clearly determined by TTE in pediatric patients with an excellent acoustic window. Balloon sizing (stop-flow method) was not performed, since it was rarely used for percutaneous ASD closure in pediatric patients in our institution. On the basis of 2D and color Doppler TTE measurement, a device 4–8 mm larger than the defect size was selected to close the defect. For defects with sufficient rims, the device was selected to be 4–6 mm larger than the defect size. For defects with floppy rims or multiple defects with the intention to cover most of the atrial septum with a single device, a larger device (7-8 mm larger than the defect size) was considered. In general, we chose the size of a PLLA device which was 2 mm larger than that of a metal device for the same defect. The delivery and deployment of the device have been described in detail in our previous study [16]. In brief, after introducing a delivery sheath into the left upper pulmonary vein (LUPV), the device was inserted and advanced into the LUPV. The delivery sheath was withdrawn to the right atrium to expose the whole device in the atrium. Under both fluoroscopic and TTE guidance, the left atrial disc, the waist, and part of the right atrial disc were shaped in order to settle down into the defect. The left disc was pulled gently against the atrial septum; then, the right atrial disk was fully opened under TTE guidance while maintaining constant tension on the cable. The device and adjacent structures were examined by TTE to evaluate the presence of residual shunt, possible impairment of the atrioventricular valves, and obstruction of venous return. Once the optimal device position was confirmed, the delivery system was activated to release the device. A final TTE was performed to demonstrate the position of the device and any residual shunt (Figure 3).

### 2.5. Follow-Up

Clinical follow-up was performed at 24 hr, 1 month, 3 months, 6 months, 12 months, 24 months, and 36 months after the procedure. At each visit, detailed 2D-TTE was performed to assess device position and residual shunt, and ECG was recorded to evaluate cardiac arrhythmia. 3D-TTE was scheduled at 36 months to evaluate the device morphology and the integrity of the atrial septum. Chest radiographs were performed at 24 hr, 6, and 12 months to assess the possible displacement of the device. Blood was taken for measurement of hematologic and biochemical parameters at 3, 6, 12, 24, and 36 months' visits. All patients were prescribed aspirin (3–5 mg/kg once daily) for 6 months after the implant procedure.

### 2.6. Outcome Measures

The primary endpoint was a composite clinical success, comprising of clinical closure success and safety at the 36-month follow-up evaluation. The criteria for clinical closure success are (1) technical success (successful deployment and retention of the device) and (2) study device retained, with clinically insignificant or no residual shunt closure.

The criteria for safety are (1) no serious adverse event related to the device or procedure through the 36-month follow-up and (2) no device embolization or reintervention to the target defect.

Secondary endpoints included technical success, procedure success (technical success and ≤2 mm residual shunt at procedure conclusion), closure success (clinically insignificant or no residual shunt), and safety (assessment of device-related and procedure-related adverse event) at each of the follow-up visits.

Residual shunt status was classified as follows: (1) complete occlusion with no residual shunt; (2) a clinically insignificant shunt was defined as a residual shunt ≤4.0 mm (determined by 2D and color-Doppler TTE) accompanied by resolution of right ventricular enlargement; or (3) a clinically significant shunt was defined as a residual shunt >4.0 mm with right ventricular enlargement. A residual shunt detected by TTE was graded as mild (≤2.0 mm), moderate (2.1–4.0 mm), and large (>4.0 mm) [17].

### 2.7. Statistical Analysis

Continuous variables with normal distribution are expressed as mean ± SD and median with interquartile range for those that were not normally distributed. Categorical variables are expressed as numbers (percentage). Comparisons between groups were performed using the chi-square or Fisher's exact test for categorical variables. Results were considered statistically significant at a *p* value <0.05.

## 3. Results

### 3.1. Study Population and Procedure Data

14 pediatric patients were screened for inclusion and 5 patients who met the criteria were eventually enrolled. The median age of the 5 patients was 3.6 years (range, 3.1–6.5 years), and the median body weight was 14.5 kg (range, 10.0–23.5 kg). Study subjects were predominately male (80%). The indication for ASD closure was the presence of right ventricle enlargement with a significant left-to-right shunt in all of the 5 patients. The mean baseline defect size was (13.0 ± 2.8) mm, and the mean ASD diameter measured during the procedure was (13.7 ± 2.9) mm. The mean ASD diameter/body surface area was (21.33 ± 6.44) mm/m^2^, and 2 of 5 patients had a relatively large defect (≥20 mm/m^2^). Patient demographics and clinical parameters for the 5 patients are summarized in Table 1.

During the procedure, a single PLLA device was attempted and was successfully implanted in all the 5 patients without periprocedural complications related to the device or delivery system. The technical success rate was 100% (5/5). The same device was retrieved and redeployed in patients no. 1 and no. 2, mainly due to perceived device instability or poor positioning. The median defect-to-device ratio was 1.33 (range, 1.28 to 1.75). The mean procedure time was (36.2 ± 11.3) min, and the mean fluoroscopy time was (6.4 ± 1.0) min. Procedure-related data are outlined in Table 2.

All 5 patients implanted with the PLLA device had demonstrated excellent conformability to the anatomy noted on TTE, and the immediate closure success rate was 100% (5/5). No adverse events were observed during and after the implantation like device embolization, thromboembolism, or vascular complications. Sinus rhythm persisted in all patients. A TTE before discharge showed the correct position of the device with no significant residual shunt, interference with the adjacent cardiac structures, or obstruction of intracardiac structures. All patients were discharged home the second day after the procedure.

### 3.2. Follow-Up Results

#### 3.2.1. Closure Success

**Figure 4:**
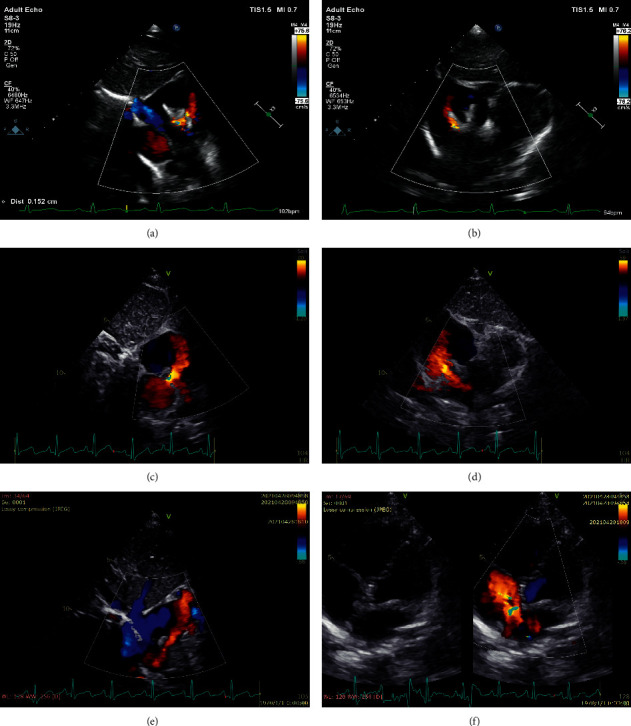
The development of residual shunt in Patient no. 2. (a-b) A mild residual shunt of 1 mm was detected at the 12-month visit. (c-d) A moderate residual shunt of 3.7 mm was detected at the 24-month visit. The device was partly degraded. (e-f) a large residual shunt of 4.3 mm was detected at the 36-month visit. The device was mostly degraded.

All 5 patients have completed the 36-month follow-up. Complete closure of the defect was achieved in 4 (80%) patients at 6 months, 3 (60%) patients at 12 months, and 2 (40%) patients at 24 and 36 months. At 24-month follow-up, as the devices have been partly degraded, 3 of the 5 patients (Patients no. 1, no. 2, and no. 3) developed new-onset residual shunts, and 2 of them (Patients no. 1 and no. 2) reached moderate degree. At 36-month follow-up, as most parts of the devices had been degraded, the residual shunt size increased over time in all the 3 patients. On TTE, the residual shunts in the 3 cases were located at the anterosuperior site of the atrial septum, close to the retroaortic rim and superior vena cava rim. In the case of Patient no. 2 (3.1 years old, male) who had a 14.9 mm defect and was implanted with a 20 mm device, a new-onset mild residual shunt of 1.5 mm was demonstrated by TTE at 1-year follow-up. The size of the residual shunt reached 4.3 mm with right ventricular enlargement at 36-month follow-up (Figure 4). Therefore, the case of Patient no. 2 was considered a clinical closure failure. Overall, the clinical closure success rate at 36 months was 80% (4/5) (Table 3).

#### 3.2.2. Right Atrial Disc Malformation

**Figure 5:**
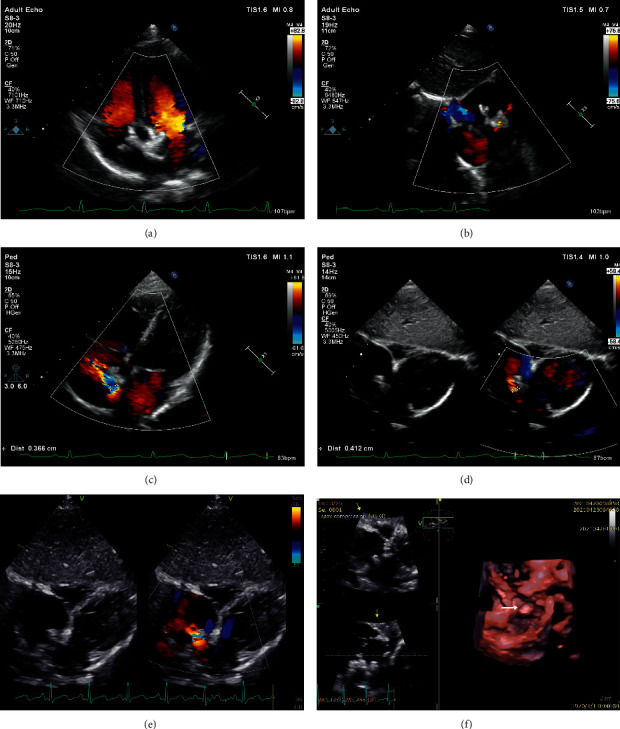
Device deformation in Patient no. 2. (a-b) TTE at the 12-month visit demonstrated that the right disc expanded to the right atrium. (c-d) TTE at the 24-month visit demonstrated that, as the device was partly degraded, a new onset residual shunt appeared. (e-f) TTE at the 36-month visit demonstrated the shrinkage size of the device and increased size of the residual shunt (e). 3D-TTE showed that the right disc apposition and incorporation into adjacent myocardium were not completed (f). White arrow indicated the remaining part of the right disc.

In the case of Patient no. 2, right atrial disc malformation was detected since the 12-month follow-up. The device was not capable of conforming well to the atrial septum due to expansion of the right atrial disc. As the degradation progress proceeded and the size of the device decreased overtime, the device could no longer maintain a normal flattened profile and a new-onset residual shunt appeared. 3D-TTE showed that the right disc apposition and incorporation into adjacent myocardium were not yet completed at 36-month follow-up (Figure 5). Patient no. 2 was under close follow-up and was free from reintervention for the defect as yet.

#### 3.2.3. Adverse Events

For the remaining 4 patients, follow-up results confirmed the safety of the device, with no device embolization, reinterventions, vascular complications, documented arrhythmias, and an excellent device shape conforming well to the septum without clinical sequelae.

The results of analyses of hematologic and biochemical markers are given in Table S1. There was no evidence of a systemic adverse response in any patient.

## 4. Discussion

Percutaneous ASD closure with complete biodegradable septal occlusion devices has emerged as an appealing strategy, for the potential of minimizing the possibilities for late complications with metal devices and preserving access to the left atrium. This article was the first to demonstrate the long-term safety and efficacy profile of the PLLA device for the treatment of secundum ASD in humans. There were no short- and long-term complications related to the device or the procedure, providing preliminary evidence of the long-term safety of the device. However, new-onset residual shunts at long-term follow-up suggested that further improvement of the long-term efficacy of the device was needed.

### 4.1. Technique Aspects

One of the main challenges of the closure technique with the PLLA device is the position and deployment process, due to the fact that the PLLA framework and membrane are all invisible on fluoroscopy. As a result, the PLLA device should be deployed under both fluoroscopic and echocardiographic guidance. TTE/TEE is mainly used to evaluate the shape and position of the device during the deployment process. During the releasing process, the device must be confirmed to be detached from the locking system, which can only be determined by fluoroscopic guidance.

The Absnow^TM^ device system has a simple design that permits precise positioning with a single handle control. By combining both fluoroscopic and echocardiographic guidance, this system provides easy and intuitive positioning during the procedure. Redeployment occurred in the first two patients, as the operators were inexperienced with the new implantation techniques. In spite of this, we observed a fast learning curve, as depicted by the short mean fluoroscopic time (6.4 minutes).

Although sizing recommendations for the PLLA device are still being developed, we tend to follow a strategy in which the device is 4–8 mm larger than the defect size (determined by 2D and color Doppler TTE during the procedure) for optimal closure. Because the PLLA device is much softer than the metal devices, we consider it is suitable to choose a PLLA device size 2 mm larger than that of a metal device for the same defect. The median device-to-defect ratio was 1.33 (range 1.28 to 1.75) in our study. Factors such as tissue rims and defect shape may have resulted in a larger sizing ratio but are still in the acceptable range.

To be noted, based on the softness of the PLLA matrix, the PLLA device may have increased risk of device instability. Moreover, the self-centrality of the PLLA device is weak. Therefore, after the right disc has been shaped, it must be pushed towards the septum with the cable, while maintaining the left disc firmly at the left side of the defect to ensure adequate contact with the septum.

### 4.2. Device Efficacy

The present study demonstrated excellent immediate- and short-term closure rates of percutaneous ASD closure using the PLLA device. However, the closure rate decreased thereafter and at 36-month follow-up, TTE proved that 2 patients developed moderate degree residual shunt and 1 patient considered closure failure. A potential concern raised by this analysis is that the long-term efficacy of the PLLA device is inferior to that of metal devices. According to our preclinical studies, the PLLA materials were almost totally degraded, and the implantation site was fully covered by a tight layer of endothelial tissue after 36 months [18]. While in a clinical study, as the PLLA devices had been mostly degraded, new tissue could not fully cover the anterosuperior site of the atrial septum, resulting in new-onset residual shunts. We assumed that the mechanism of incomplete closure may be related to two aspects: the design and nature of the PLLA device and the structure of the atrial septum and defect.

When at a locked state in vitro, the two discs of the device are tightly fixed together towards the center. However, the softness of the PLLA matrix diminishes the resilience force of the device framework, reducing the device's ability to maintain the original structural features. Repeated operation on the device in vivo would cause significant strain to the device structure and decrease its self-accommodation inside the atrial septum. The waist height is actually larger than that of the original structure, resulting in a larger distance between the two discs. In cases of patients with young age, thin atrial septum, deficient rims, and repeated deployment attempts, the two discs would not be able to adequately affix to the atrial septum, leaving a gap between the device and the septum. The new endothelial tissue could not “climb over” the gap to fully repair the defect and eventually contribute to incomplete healing of the defect and potential closure failure.

Previous studies focusing on a biodegradable device such as the BioStar device have demonstrated that one of the drawbacks of this type of occluder was the possibility of an initially closed defect that might reappear or a residual shunt might persist and even grow after initiation of the degradation process [19, 20]. Our series proved that this possibility cannot be ruled out. However, as a pilot series, our patients were significantly challenging in terms of young age, large defect size/BSA ratio, and anatomic complexity. What is more, deployment of this device was technically more challenging than metal devices because of its weak self-centring ability. Deeper understanding of the device's advantages and drawbacks and better modifications of the device structure and deployment technique are warranted to further improve the clinical closure success.

### 4.3. Device Safety

There were no major adverse events reported in the present study, and no major safety issues related to the PLLA device were identified. This study confirms the preliminary safety of percutaneous ASD closure using the device both early and during long-term follow-up.

Device malformation with right disc expansion was detected in one case at 12-month follow-up. We assumed that incomplete endothelialization after degradation initiation due to inadequate device conformation to the atrial septum might be a possible reason. Neither thrombus formation nor systemic thromboembolism was detected in this case during follow-up. In our preclinical studies, PLLA materials were confirmed to be surrounded by fibrous capsules in both animal muscle tissues and ASD models after implantation within 3 months [18]. Similarly, the PLLA device may also be covered with fibrous tissues shortly after implantation in humans. The fibrous capsules could prevent thromboembolism of PLLA degradation pieces even in cases in which device integrity problems or device deformation occurred. Unfortunately, neither CT pulmonary angiography nor MR angiography of the brain was performed to evaluate for microembolization of PLLA fragments in this case. Further examinations would be scheduled to evaluate possible thromboembolism complications in future studies.

### 4.4. Study Limitation

We recognize that there are several limitations to our study. First, this study is limited by its small sample size and lack of previous clinical experience. Second, the size of the defects and the residual shunt was evaluated by 2D and color Doppler TTE in our study, which may underestimate the size of the defects and the residual shunt. A multicenter registry (NCT03601039) has been launched in August 2018 in China mainland to provide more evidence of the Absnow^TM^ device for percutaneous ASD closure.

## 5. Conclusion

The present study proved the preliminary safety of the Absnow^TM^ device in percutaneous closure of secundum ASD during a follow-up period of 3 years. Despite the high complete closure rate at short-term follow-up, new-onset residual shunt played a significant role in diminishing its long-term efficacy. A precise selection of suitable patients based on age, ASD size, and morphology; a selection of a device of an appropriate size; and improvement of device structure and deployment technique may help achieve a higher closure rate. Large multicenter studies are required to further establish the safety and efficacy profile of the Absnow^TM^ device.

## Figures and Tables

**Table 1:** Patient demographics.

	Mean (SD)	Median	Range	Patient no. 1	Patient no. 2	Patient no. 3	Patient no. 4	Patient no. 5
Age (years)	4.3 (1.3)	3.6	(3.1, 6.5)	4.9	3.1	6.5	3.2	3.6
Gender	—	—	—	Male	Male	Male	Female	Male
Weight (kg)	16.3 (5.1)	14.5	(10, 23.5)	23.5	10.0	21.0	14.5	12.5
Body surface area (m^2^)	0.67 (0.18)	0.61	(0.45, 0.92)	0.92	0.45	0.84	0.61	0.54
Baseline ASD size (mm)	13.0 (2.8)	14.0	(8.0, 16.0)	16.0	14.0	15.0	12.0	8.0
ASD size at procedure (mm)	13.7 (2.9)	14.9	(8.0, 16.5)	16.5	14.9	15.0	14.0	8.0
ASD size/BSA (mm/m^2^)	21.33 (6.44)	17.93	(14.81, 33.11)	17.93	33.11	17.86	22.95	14.81

*Abbreviations*. SD: standard deviation; ASD: atrial septal defect; BSA: body surface area.

**Table 2:** Procedure data.

	Mean (SD)	Median	Range	Patient no. 1	Patient no. 2	Patient no. 3	Patient no. 4	Patient no. 5
Device size (mm)	—	20	(14, 24)	24	20	20	18	14
Sheath (French)	—	12	(10, 12)	12	12	12	10	10
Device-to-defect size	1.43 (0.17)	1.33	(1.28, 1.75)	1.45	1.34	1.33	1.28	1.75
Procedure time (minute)	36.2 (11.3)	31	(28, 52)	52	26	31	44	28
Fluoroscopic time (minute)	6.4 (1.0)	6.0	(5.4, 7.6)	7.6	5.4	6.0	7.3	5.5
Qp : Qs	1.7 (0.2) : 1	1.7 : 1	(1.5 : 1, 2.0 : 1)	1.7 : 1	2.0 : 1	1.8 : 1	1.5 : 1	1.5 : 1

**Table 3:** Residual shunts and clinical significance.

All patients (*n* = 5)	1 month	6 months	12 months	24 months	36 months
Technical success	*N* = 5	*N* = 5	*N* = 5	*N* = 5	*N* = 5
Clinical closure success	5/5 (100%)	5/5 (100%)	5/5 (100%)	5/5 (100%)	4/5 (80%)
Complete occluded	4/5 (80%)	4/5 (80%)	3/5 (60%)	2/5 (40%)	2/5 (40%)
Clinical insignificant shunt	1/5 (20%)	1/5 (20%)	2/5 (40%)	3/5 (60%)	2/5 (40%)
Clinical significant shunt	0	0	0	0	1/5 (20%)
Measured closure success	5/5 (100%)	5/5 (100%)	5/5 (100%)	5/5 (100%)	4/5 (80%)
Complete occluded	4/5 (80%)	4/5 (80%)	3/5 (60%)	2/5 (40%)	2/5 (40%)
0–2.0 mm residual shunt	1/5 (20%)	0	1/5 (20%)	1/5 (20%)	0
2.1–4.0 mm residual shunt with normalization of right heart parameters	0	1/5 (20%)	1/5 (20%)	2/5 (40%)	2/5 (40%)
Measured closure failure	0	0	0	0	1/5 (20%)
>4.0 mm residual shunt	0	0	0	0	1/5 (20%)

## Data Availability

Data are available on reasonable request due to privacy/ethical restrictions.
