# Supplementary material for: Initial Clinical Experience with the Biodegradable AbsnowTM Device for Percutaneous Closure of Atrial Septal Defect: A 3-Year Follow-Up
Source: J Interv Cardiol. 2021 Jul 30;2021:6369493. doi: 10.1155/2021/6369493 (PMC8349294; doi:10.1155/2021/6369493)
Supplement: Supplementary Materials — Levels of Hematologic and biochemical markers are provided in Table S1 in the Supplementary Material. [file 6369493.f1.docx]

**Table S1. Levels of Hematologic and biochemical markers (Mean±SD)**

|  | **Baseline** | **3 months** | **6 months** | **12 months** | **24 months** | **36 months** |
| --- | --- | --- | --- | --- | --- | --- |
| **WBC,10^9^/L** | 8.01±2.11 | 9.08±1.02 | 8.04±1.86 | 7.60±2.70 | 6.66±1.03 | 6.79±0.67 |
| **PLT,10^9^/L** | 291.8±89.3 | 313.6±70.0 | 365.2±107.8 | 385.2±113.0 | 328.4±58.2 | 296.8±24.8 |
| **Neu,10^9^/L** | 2.81±1.43 | 4.21±0.44 | 3.68±1.13 | 3.12±1.86 | 2.68±0.73 | 2.65±0.52 |
| **Lym,10^9^/L** | 4.27±1.31 | 3.76±0.73 | 3.57±0.95 | 3.61±0.68 | 3.28±0.35 | 3.27±0.50 |
| **CK，U/L** | 122.8±37.2 | 124.6±52.5 | 110.2±28.2 | 90.60±25.5 | 112.2±33.7 | 115.6±31.8 |
| **CK-MB，U/L** | 23.70±2.99 | 28.78±10.11 | 35.28±14.97 | 25.36±2.41 | 21.14±4.16 | 20.50±5.20 |
| **LDH，umol/L** | 258.6±43.1 | 296.6±136.5 | 294.6±70.8 | 230.8±53.1 | 241.2±54.8 | 211.0±34.4 |
| **UA，U/L** | 310.4±62.4 | 305.4±30.4 | 350.5±60.8 | 321.2±95.2 | 342.0±63.9 | 349.5±99.1 |

Abbreviations: SD, standard deviation; WBC, white blood cell; PLT, platelet; Neu, Neutropil; Lym,lymphocyte; CK, creatine kinase; CK-MB, creatine kinase-MB; LDH, lactate dehydrogenase; UA, uric acid.
